# Perceived impact of the patent linkage system on pharmaceutical market from the viewpoint of the domestic manufacturers in South Korea

**DOI:** 10.1186/s12992-022-00829-1

**Published:** 2022-03-21

**Authors:** Nahye Choi, Kyung-Bok Son, Joonsoo Byun, Dong-Wook Yang

**Affiliations:** 1 Department of Public Health Science, Graduate School of Public Health, Seoul National University, Seoul, South Korea; 2 College of Pharmacy, Hanyang University, Ansan, Gyeonggi-do, South Korea; 3 National Cancer Center, Goyang, Gyeonggi-do South Korea; 4 School of Pharmacy, Sungkyunkwan University, Suwon, Gyeonggi-do, South Korea

**Keywords:** Patent linkage, Perception, Manufacturer, Policy evaluation, South Korea

## Abstract

**Background:**

The United States requires a patent linkage system in other countries as part of free trade agreements. However, introducing a patent linkage system could be a significant barrier to the timely approval of generic drugs. This study aimed to evaluate the perceived impact of the patent linkage system in South Korea held by domestic manufacturers and analyze variations in evaluating the system according to the characteristics of domestic manufacturers.

**Methods:**

In 2020, we conducted a questionnaire survey of 39 domestic manufacturers. The survey consisted of perceptions of the system, factors affecting patent challenges, and the perceived impact of the system. A 5-point Likert scale was used to rate each item. Domestic manufacturers were categorized into three groups based on their experience of listing a patent and acquiring first generic exclusivity.

**Results:**

More than half of the manufacturers surveyed had experience of listing a patent. The patent linkage system could protect the involved patents. However, manufacturers perceived that they could successfully challenge the validity of the involved patents and then obtained market approval for generic drugs. Manufacturers responded that market size, expectations for succeeding in litigation, and expectations for manufacturing the drug were the most relevant factors when they initiated patent challenges. Manufacturers reported that the system, in particular the first generic exclusivity, enhanced the research and development capability of generic manufacturers, increased their domestic sales, and improved access to generic drugs.

**Conclusions:**

The perceived impact of the patent linkage system was limited to the domestic market and generic drugs. In narrowing the impact to the effects on the domestic industry, the system had positive impacts of the system on generic manufacturers. The first generic drug exclusivity lies at the center of this positive perception. However, manufacturers perceived that the current system did not provide enough incentives for domestic manufacturers to be granted first generic drug exclusivity through patent challenges.

**Supplementary Information:**

The online version contains supplementary material available at 10.1186/s12992-022-00829-1.

## Introduction

The patent linkage system links marketing approval for a generic drug with the patent status of its reference brand-name drug [1, 2]. A generic manufacturer cannot obtain market approval if patent litigation is initiated by the manufacturer of a brand-name drug and/or the patent holder of a brand-name drug [3, 4]. The United States (U.S.) established the patent linkage system in 1984 through the Drug Price Competition and Term Restoration Act of 1984 [5, 6]. The U.S. has requested a patent linkage system in other countries as part of free trade agreements (FTAs). Canada, Australia, and South Korea introduced such systems in 1993, 2005, and 2012, respectively [7, 8].

The case in the U.S. is known as an exemplar in understanding the system [5]. The patent linkage system in the U.S. is composed of four parts, the patent list, notification process, a stay of generic approval, and first generic exclusivity [4, 5, 7, 9]. When a manufacturer seeks market approval for a new chemical drug (new drug), the manufacturer lists relevant patents associated with the new drug in the Orange Book. Another manufacturer seeking market approval for a generic drug must assert that the relevant patent in the Orange Book is invalid or will not be infringed upon, a so-called *paragraph iv certification*, and notifies the manufacturer of the new drug of a *paragraph iv certification*. The manufacturer of a new drug can initiate infringement litigation and then request that the Food and Drug Administration (FDA) impose a stay of generic approval of up to 30 months. In contrast, the FDA could grant 180-day exclusivity for the first manufacturer to file a successful *paragraph iv certification*. The FDA cannot approve another generic version of the new drug during this 30-month period.

Consistent with the case in the U.S., the system in South Korea also has four parts [10–12]. The patent list and the notification process were implemented in March 2012. The stay of generic approval and first generic exclusivity were introduced in March 2015. The patent linkage system provides early dispute resolution for patent infringement before the generic drug that might infringe upon the relevant patent enters the market [13]. In particular, the regulatory authority undertakes the stay of generic approval based on the infringement litigation initiated by the manufacturer of a new drug and/or patent holder for a new drug [14]. Under the system, generic manufacturers are concerned about patent issues in addition to the safety, efficacy, and quality of a generic when seeking market approval [10–12]. Thus, the patent linkage system could be a significant barrier to the timely approval of generic drugs [15–17]. Many researchers and civic activists in South Korea have warned that the system could cause delayed generic drug entrance and strengthen the monopoly of a new drug [18, 19].

The regulatory process for granting market approval for generic drugs has been significantly changed by the patent linkage system [20, 21]. Domestic manufacturers that introduce generics to the market are the main party affected by the system [18, 19]. However, their perceptions of the system and business strategies after introducing the system have not yet been reported. This study aimed to evaluate the perceived impact of the patent linkage system in South Korea held by domestic manufacturers and analyze variations in evaluating the system according to the characteristics of domestic manufacturers. To this end, we developed a questionnaire survey for domestic manufacturers to understand their perceptions of the system, factors affecting patent challenges, and their perceived impact of the system.

## Methods

### Study design

We recruited participants for the survey through the cooperation of the Korea Pharmaceutical Patent Institution (KPPI) and the Korea Pharmaceutical and Bio-Pharma Manufacturers Association (KPBMA). The KPPI and KPBMA forwarded information on this survey to their members. A total of 39 domestic manufacturers responded to the survey from September 28, 2020, to October 14, 2020. This survey was approved by the Institutional Review Board (IRB) of Ewha Woman’s University (IRB No. ewha-202009–0028-01).

**Fig. 1:**
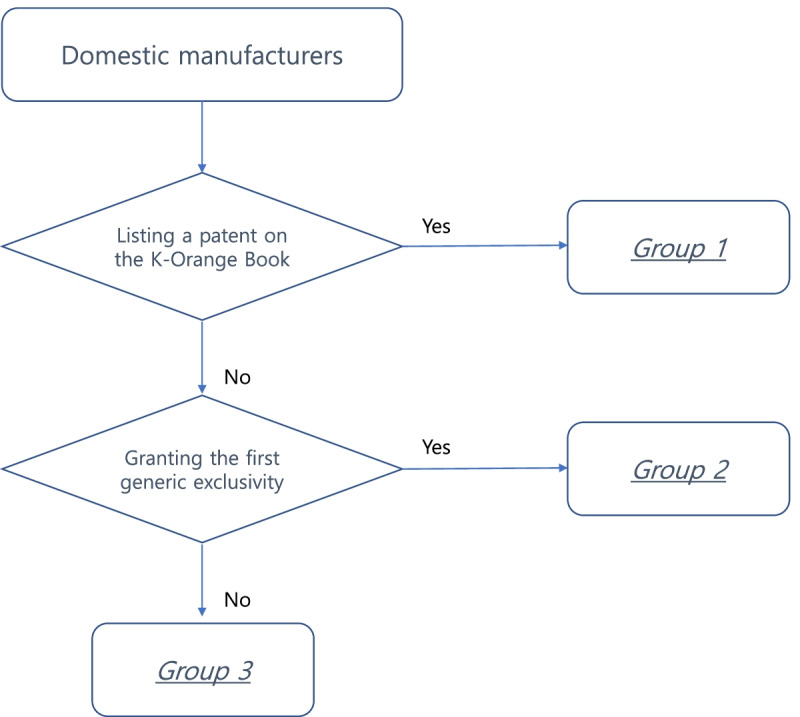
Categorization of the manufacturers into three groups

Figure 1 describes the process of categorizing the manufacturers. Based on the experience of listing a patent in the K-Orange Book and being granted first generic exclusivity, we categorized domestic manufacturers into three groups. Group 1 manufacturers had a patent listed in the K-Orange Book. Note that manufacturers belonging to group 1 could be also granted first generic exclusivity after a successful patent challenge. Group 2 manufacturers did not have a patent listed in the K-Orange Book. However, they were granted first generic exclusivity after a successful patent challenge. Group 3 manufacturers did not have a patent listed in the K-Orange Book and were not granted first generic exclusivity. Thus, group 1 represented manufacturers that had produced patented drugs and had successfully utilized the patent linkage system to be granted first generic entrant status, group 2 represented follow-on manufacturers that had successfully utilized the patent linkage system to be granted first generic entrant status, and group 3 represented follow-on manufacturers that had not utilized the patent linkage system.

### Survey questionnaire

The survey questionnaire consisted of three sections: perceptions of the system, factors affecting patent challenges, and the perceived impact of the system. The first section collected the perceptions of the system held by domestic manufacturers. In particular, we chose two parts of the system, the stay of generic market approval and 9-month exclusivity for the first generic entrant. The second section asked manufacturers about the factors affecting patent challenges. The patent linkage system upgrades patent challenges for domestic manufacturers to be granted timely market approval for generics. We provided eight factors and asked about their relevance in initiating patent challenges. The third section asked about the perceived impact of the system in terms of research and development capability, pharmaceutical sales, accessibility, and employment. We used a 5-point Likert scale (from -2 for never relevant to 2 for very relevant) to rate each item. The survey results are presented as average values and variances. The survey questionnaire is described in Supplementary 1.

## Results

### Characteristics of the manufacturers

Table 1 presents the manufacturers’ characteristics. A total of 39 manufacturers responded to the survey. Of them, 21 (54%), 11 (28%), and 7 (18%) were categorized into group 1, group 2, and group 3, respectively. We requested information about the manufacturer in terms of annual sales, research and development intensity (R&D intensity), designation as an innovative pharmaceutical manufacturer, and experience in developing new drugs or modified new drugs. Note that the Ministry of Health and Welfare designates research-based manufacturers as innovative manufacturers. As of December 2020, 48 manufacturers were designated as innovative manufacturers [22]. The annual sales and R&D intensity were in the order of group 1, group 2, and group 3, implying that manufacturers belonging to group 1 were major (or big-sized) manufacturers based on financial resources and investment in research and development. Similarly, manufacturers belonging to group 1 were more likely to be designated as innovative manufacturers and develop new drugs and modify new drugs than manufacturers belonging to groups 2 or 3.

**Table 1:** Characteristics of the manufacturers

	Total (*n* = 39)	Group 1 (*n* = 21)	Group 2 (*n* = 11)	Group 3 (*n* = 7)
**Annual sales (million KRW)**				
~ 100,000	12 (30.8%)	0 (0%)	6 (54.5%)	6 (85.7%)
100,000 ~ 300,000	13 (33.3%)	7 (33.3%)	5 (45.5%)	1 (14.3%)
300,000 ~	14 (35.9%)	14 (66.7%)	0 (0%)	0 (0%)
**Research and development intensity**				
~ 5%	10 (25.6%)	3 (14.3%)	3 (27.3%)	4 (57.1%)
5% ~ 7%	10 (25.6%)	6 (28.6%)	2 (18.2%)	2 (28.6%)
7% ~ 10%	10 (25.6%)	6 (28.6%)	4 (36.4%)	0 (0%)
10% ~	9 (23.1%)	6 (28.6%)	2 (18.2%)	1 (14.3%)
**Designation as an innovative pharmaceutical manufacturer**				
Yes	14 (35.9%)	12 (57.1%)	2 (18.2%)	0 (0%)
No	25 (64.1%)	9 (42.9%)	9 (81.8%)	7 (100%)
**Experience in developing new drugs**				
Yes	16 (41.0%)	13 (61.9%)	1 (9.1%)	2 (28.6%)
No	23 (59.0%)	8 (38.1%)	10 (90.9%)	5 (71.4%)
**Experience in developing modified new drugs**				
Yes	27 (69.2%)	17 (81.0%)	7 (63.6%)	3 (42.9%)
No	12 (30.8%)	4 (19.0%)	4 (36.4%)	4 (57.1%)
**Experience in granting first generic exclusivity after a successful patent challenge**				
Yes	30 (76.9%)	19 (90.5%)	11 (100%)	0 (100%)
No	9 (23.1%)	2 (9.5%)	0 (0%)	7 (100%)

Manufacturers belonging to group 1 had a patent listed in the K-Orange Book. Manufacturers belonging to group 2 did not have a patent listed in the K-Orange Book. However, they were granted first generic exclusivity after a successful patent challenge. Manufacturers belonging to group 3 did not have a patent listed in the K-Orange Book and were not granted first generic exclusivity

### Perceptions of the patent linkage system

**Fig. 2:**
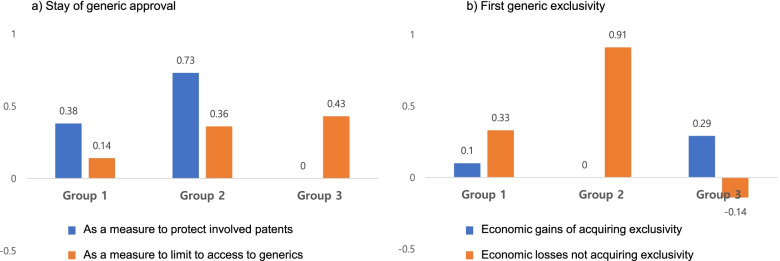
Perceptions of the patent linkage system

Figure 2 describes the perceptions of the patent linkage system held by domestic manufacturers. In particular, we selected two parts of the system, the stay of generics and first generic exclusivity, and asked the respondents’ opinions on these two parts. Manufacturers rated the items on the stay of generics as a measure to protect the involved patents and limit access to generic drugs with 0.41 points (pts) and 0.26 pts, respectively. However, the score varied when we categorized the manufactures into three groups. “To protect patents” was rated higher than “to limit access to generics” by group 1 and group 2 (0.38 vs. 0.14 and 0.73 vs. 0.36, respectively). In contrast, “to limit access to generics” was rated higher than “to protect patents” by group 3 (0.43 vs. 0.00). We also asked about the economic gains of acquiring first generic exclusivity and the economic losses of not acquiring first generic exclusivity. Manufacturers rated “economic losses” and “economic gains” with 0.41 pts and 0.10 pts, respectively. However, the score also varied according to the group. “Economic losses” were rated higher than “economic gains” by group 1 and group 2 (0.33 vs. 0.10 and 0.91 vs. 0.00, respectively). In contrast, “economic gains” were rated higher than “economic losses” by group 3 (0.29 vs. -0.14).

### Factors affecting patent challenges

Table 2 presents the factors affecting patent challenges. All factors except for the expected cost of litigation scored higher than 0 pts. Overall, domestic manufacturers responded that market size, expectations for succeeding in litigation, and expectations for manufacturing the drug were relevant factors (rated more than 1 pts) when they initiated patent challenges. However, each group identified different factors as being relevant. Group 1 responded that market size and expectation for succeeding in litigation were relevant factors. Group 2 responded that market size, expectation for manufacturing, expectation for collaboration, expectation for succeeding in litigation, and sales force were relevant factors. Group 3 responded that market size was the only relevant factor.

**Table 2:** Perceptions of factors affecting the initiation of patent challenges

	Total	Group 1	Group 2	Group 3
Market size of the originator drug	1.56 (0.63)	1.52 (0.66)	1.82 (0.39)	1.29 (0.70)
Expectations for succeeding in litigation	1.18 (0.90)	1.43 (0.58)	1.09 (1.00)	0.57 (1.18)
Expectations for manufacturing a generic drug	1.03 (0.80)	0.90 (0.92)	1.27 (0.62)	1.00 (0.53)
Sales force of a manufacturer	0.87 (0.82)	0.86 (0.77)	1.09 (0.90)	0.57 (0.73)
Market power of a manufacture	0.85 (0.74)	1.00 (0.62)	0.45 (0.89)	1.00 (0.53)
Product portfolios of a manufacturer	0.79 (0.76)	1.00 (0.69)	0.73 (0.86)	0.29 (0.45)
Expectations for collaboration with other manufacturers	0.69 (0.99)	0.62 (0.90)	1.18 (0.94)	0.14 (0.99)
Expected cost of a court litigation	-0.13 (0.88)	-0.29 (0.76)	0.00 (1.04)	0.14 (0.83)

### Perceived impact of the patent linkage system

Table 3 describes the perceived impact of the patent linkage system in terms of research and development, sales, access to medicine, and employment. Manufacturers agreed with the fact that the system improved research and development capability for modified new drugs, the first generic, and patent analysis. They responded that the system improved access to generic drugs and increased their domestic sales. However, manufacturers disagreed with the fact that the system enhanced research and development capability for new drugs, improved access to new drugs, and increased overseas sales. Finally, the manufacturers responded that the system caused an increase in employment in the departments of patent analysis, research and development, and regulatory affairs.

**Table 3:** Perceived impact of patent linkage system in selected areas

	Total	Group 1	Group 2	Group 3
**Enhanced research and development capability**				
Research on new drugs	-0.54 (0.98)	-0.62 (1.05)	-0.64 (0.88)	-0.14 (0.83)
Research on modified new drugs	0.46 (1.06)	0.43 (1.00)	0.55 (1.30)	0.43 (0.73)
Research on the first generic	1.18 (0.75)	1.05 (0.65)	1.36 (0.98)	1.29 (0.45)
Analyze related patents	0.79 (0.91)	0.71 (0.76)	1.18 (0.94)	0.43 (1.05)
**Increased sales**				
Domestic sales	0.33 (0.69)	0.24 (0.68)	0.36 (0.64)	0.57 (0.73)
Overseas sales	-0.90 (0.84)	-1.10 (0.75)	-0.82 (0.83)	-0.43 (0.9)
**Improved access to medicines**				
Access to new drugs	-0.18 (0.96)	-0.14 (1.08)	-0.36 (0.64)	0.00 (0.93)
Access to generic drugs	0.38 (0.89)	0.67 (0.78)	-0.09 (0.79)	0.29 (1.03)
**Increased employment**				
Patent department	0.46 (1.01)	0.29 (0.70)	0.73 (1.21)	0.57 (1.29)
Research and development department	0.26 (0.78)	0.10 (0.68)	0.55 (0.89)	0.29 (0.70)
Regulatory affairs department	0.23 (0.80)	0.05 (0.72)	0.45 (0.99)	0.43 (0.49)
Pricing and reimbursement department	-0.26 (0.90)	-0.24 (0.68)	-0.27 (1.35)	-0.29 (0.45)
Sales and marketing department	-0.15 (0.83)	-0.29 (0.76)	0.00 (1.04)	0.00 (0.53)

## Discussion

This study aimed to evaluate the perceived impact of the patent linkage system in South Korea from the viewpoint of domestic manufacturers. To this end, we developed a survey questionnaire on the perceptions of the system, factors affecting patent challenges, and the perceived impact of the system. This was the first study that comprehensively evaluated the perceived impact of the patent linkage system according to domestic manufacturers. Findings from this study could further our understanding of the patent linkage system in the context of the domestic pharmaceutical industry.

### Variations in evaluating the system

We categorized domestic manufacturers into three groups to present variations in evaluating the patent linkage system. Group 1 represented manufacturers that had produced patented drugs and had successfully utilized the patent linkage system to be granted first generic entrant status. Groups 2 and 3 represented manufacturers who had not yet produced patented drugs. Manufacturers belonging to groups 2 and 3 had produced generic drugs. However, the difference between group 2 and group 3 is noteworthy. Manufacturers belonging to group 2 had succeeded in utilizing the patent linkage system and introduced generic drugs after challenging the related patents. In contrast, manufacturers belonging to group 3 had not succeeded in utilizing the system. We assumed that group 1 took the “originator” and “generic” stance toward the system and group 2 took the “generic” stance toward the system with capturing an advantage, in particular first generic exclusivity. However, group 3 took the “generic” stance toward the system without capturing an advantage.

Such grouping helped us to comprehensively understand the impact of the patent linkage system. We analyzed the perceptions of the manufacturers toward a stay of generic approval and first generic exclusivity. First, a stay of generic is caused by the action of an originator for patent infringement. Market approval for a generic drug is delayed for 9 months if the litigation is not concluded in the generic manufacturer’s favor. Next, first generic exclusivity is granted for manufacturers who successfully challenge the validity of the involved patents. Manufacturers belonging to group 3 responded differently than those belonging to groups 1 and 2. For instance, “limit access to generics” was rated higher than “protect patents” by group 3 in terms of a stay of generic. “Economic gains” were also rated higher than “economic losses” by group 3 in terms of acquiring first generic exclusivity. Such different responses could be explained by the lack of successful experience of the manufacturers in group 3. Furthermore, manufacturers belonging to group 3 did not have enough human or financial resources to initiate patent challenges.

The responses from the manufacturers in groups 1 and 2 were noteworthy. They agreed with the fact that a stay of generic approval was a measure to protect the involved patents. However, the degree of agreement was decreased when we asked whether a stay of generic approval was a measure to limit access to generic drugs. These findings indicated that the patent linkage system could protect the involved patents. However, manufacturers perceived that they could successfully challenge the validity of the involved patents, and then, they could be granted market approval for generics. Similarly, we asked about the economic gains and losses associated with acquiring first generic exclusivity. They responded that the economic gains of acquiring first generic exclusivity were marginal. However, the score was increased when we asked about the economic losses of not acquiring first generic exclusivity. These findings implied that the current system does not provide enough incentives for domestic manufacturers to be granted first generic exclusivity through patent challenges.

### Determinants of patent challenges

We provided factors affecting patent challenges. It has been well documented that a generic manufacturer is more likely to challenge the validity of patented drugs with higher sales [23–27]. In this study, the market size of the originator drug was the most influential factor in deciding to initiate patent challenges. The cost of litigation and expectations for succeeding in litigation were other factors associated with patent challenges [28]. However, the expected cost of litigation was not an influential factor for domestic manufacturers in South Korea. Manufacturers rated the relevance of cost of litigation as -0.13, which was the lowest score among the eight factors. This finding might be associated with the current system for patent challenges. The litigation system in South Korea is composed of judicial courts and the intellectual property tribunal (IPT), in a so-called two-tiered litigation system [29]. The cases of damages and injunctions against patent infringement are covered by the judicial courts, while cases involving the (in)validity of patents are covered by the IPT [29]. Domestic manufacturers mainly utilize the IPT for the (in)validity of patents [10]. The cost of litigation on the (in)validity of patents is not expensive. It was interesting that manufacturers belonging to group 3 rated the cost of litigation higher than the manufacturers belonging to groups 1 and 2. Note that manufacturers belonging to group 3 did not have enough financial resources to initiate patent challenges. We also found that manufacturers belonging to group 2 rated collaboration with other manufacturers as high. Manufacturers belonging to group 2 had experience in being granted first generic exclusivity. Some of them had utilized collaboration with other manufacturers to initiate patent challenges. This business strategy might explain their response.

### Evaluation of the system

Given the structure of the pharmaceutical market in South Korea, many researchers and civil activists have anticipated that the patent linkage system could cause detrimental effects on the local pharmaceutical industry that introduces generic drugs into the market. In the previous literature, the patent linkage system did not influence the availability of new drugs [7] nor delay the introduction of new drugs [30]. Consistent with the anticipation and the previous literature, the overall impact of the patent linkage system was limited to the domestic market and generic drugs in South Korea. We evaluated the perceived impact of the patent linkage system in terms of research and development, sales, access to medicines, and employment. The system seemed to partially improve the activity of research and development for generics, access to generics, and domestic sales. However, the impact was limited to certain types of drugs and the domestic market. For instance, the impact of the system on research activities for developing new drugs, overseas sales, and access to new drugs was not positively perceived by domestic manufacturers.

In narrowing the impact to the effects on the domestic industry, we found that the system had a positive impact on generic manufacturers. First generic exclusivity, which provides an economic incentive to challenge the validity of the involved patents, lies at the center of this positive impact. However, the economic gains from acquiring first generic exclusivity seemed to be unattractive for domestic manufacturers. The patent linkage system in South Korea guarantees exclusivity for domestic manufactures that have acquired first generic exclusivity. The 9-month exclusivity is much longer than the 180-day exclusivity given by the system in the U.S. [5]. However, generic manufacturers in South Korea responded that 9-month exclusivity did not provide enough economic incentives to initiate patent challenges.

Market penetration can give clues to interpreting this interesting finding. As already discussed, the market size of the originator drug was the most influential factor affecting patent challenges. Generic manufacturers decide whether or not to challenge patents based on the market size of the originator drug. In particular, their market penetration determines sales and/or profits during the 9-month exclusivity. However, the market penetration of generic drugs that acquired first generic exclusivity was marginal in South Korea [10–12]. Marginalized penetration implies that even if the patent linkage system in South Korea guarantees a longer exclusivity period than that in the U.S., the current market could not provide enough incentives for generic manufacturers.

### Study limitations

This study had several limitations. First, this study included 39 domestic manufacturers. Further research with larger sample size is needed to fully evaluate the system. However, the number of domestic manufacturers that can utilize the patent linkage system in South Korea is not large. A total of 77 domestic manufacturers were granted first market exclusivity from 2015 to 2019 [31]. Thus, the 39 domestic manufacturers included in this study were not too small to draw conclusions. Second, the majority of our findings were based on the perceptions of domestic manufacturers. This study conducted surveys of domestic manufacturers while members of foreign manufacturers, academia, and government authorities were excluded. Other empirical research and/or survey research including other stakeholders is needed to supplement the findings of this research. Third, this study evaluated the patent linkage system in South Korea. Thus, the findings from this research cannot be generalized to other countries with different settings and contexts. Variations in the structure of the pharmaceutical industry, market, and patent challenging system could influence the perceived impact of the system held by domestic manufacturers.

## Conclusion

The patent linkage system could protect the involved patents. However, manufacturers perceived that they could successfully challenge the validity of the involved patents, and then be granted market approval for generics. The perceived impact of the patent linkage system was limited to the domestic market and generic drugs in South Korea. In narrowing the impact to the effects on the domestic industry, the system had a positive impact on generic manufacturers. First generic exclusivity lies at the center of this positive perception. However, manufacturers perceived that the current system did not provide enough incentives for domestic manufacturers to be granted first generic exclusivity through patent challenges.

## Supplementary Information

**Additional file 1.** Survey on the impact of patent linkage system.

## Data Availability

The datasets analyzed during the current study are available from the corresponding author upon reasonable request.
