# Supplementary material for: Perceived impact of the patent linkage system on pharmaceutical market from the viewpoint of the domestic manufacturers in South Korea
Source: Global Health. 2022 Mar 21;18:34. doi: 10.1186/s12992-022-00829-1 (PMC8935685; doi:10.1186/s12992-022-00829-1)
Supplement: Supplementary file 1 — Additional file 1. Survey on the impact of patent linkage system. [file 12992_2022_829_MOESM1_ESM.pdf]

Survey ID	

Survey on the impact of patent linkage system

We are conducting a study by the Ministry of Food and Drug Safety, <Evaluation of the impact of patent linkage system>. The purpose of the study is to investigate the status of the patent linkage system in Korea, and evaluate the impact of the system. We plan to survey pharmaceutical companies' perceptions of the patent linkage system.

Participation in this study is voluntary and anonymous. You can withdraw your consent at any time. The results of the survey will be used for investigation purposes and can be used as evidence for system improvement.

Thank you for agreeing to take part in this survey.

Only one person in charge of the patent linkage system can participate in this survey.<br>If possible, please answer on behalf of your company.

The following are questions regarding the stay of generic approval in Korea.

1-1. How effective do you think the stay of generic approval is as a measure to protect the involved patents for originator drugs?

☐ Very ineffective

☐ Ineffective

☐ Moderate

☐ Effective

☐ Very effective

1-2. How effective do you think the stay of generic approval is as a measure to limit market access to generic drugs?

☐ Very ineffective

☐ Ineffective

☐ Moderate

☐ Effective

☐ Very effective

The following are questions regarding first generic exclusivity in Korea.

2-1. How much do you think your company's economic gains are from acquiring first generic exclusivity?

☐ Very low

☐ Low

☐ Moderate

☐ High

☐ Very high

2-2. How much do you think your company's economic losses are from not acquiring first generic exclusivity?

☐ Very low

☐ Low

☐ Moderate

☐ High

☐ Very high

3-1. Regarding the effectiveness of first generic exclusivity, do you think it is necessary to limit the number of drugs acquiring first generic exclusivity per item?

☐ Yes **(If yes, go to question 3-2)**

☐ No (maintain the current status) **(If no, go to question 3-3)**

3-2. How many items are appropriate for first generic exclusivity acquisition?

☐ 1

☐ 2-3

☐ 4-5

☐ 6-10

☐ ≥ 11

☐ Other (please specify)

3-3. Regarding the effectiveness of first generic exclusivity, do you think first generic exclusivity period (9 months) is appropriate?

☐ No **(If no, go to question 3-4)**

☐ Yes (maintain the current status) **(If yes, go to question 4)**

3-4. How long is the appropriate first generic exclusivity period?

☐ Shorter than the current status (- month)

☐ Longer than the current status (- month)

4. The following are the expected factors affecting the initiation of patent challenges for first generic exclusivity. How much do you think each factor is related to the initiation of patent challenges for first generic exclusivity?

4-1. Expectations for succeeding in litigation

☐ Never relevant

☐ Not very relevant

☐ Moderate

☐ Relevant

☐ Very relevant

4-2. Expected cost of a court litigation

☐ Never relevant

☐ Not very relevant

☐ Moderate

☐ Relevant

☐ Very relevant

4-3. Market size of the originator drug

☐ Never relevant

☐ Not very relevant

☐ Moderate

☐ Relevant

☐ Very relevant

4-4. Product portfolios of a manufacturer

☐ Never relevant

☐ Not very relevant

☐ Moderate

☐ Relevant

☐ Very relevant

4-5. Market power of a manufacture

☐ Never relevant

☐ Not very relevant

☐ Moderate

☐ Relevant

☐ Very relevant

4-6. Sales force of a manufacturer

☐ Never relevant

☐ Not very relevant

☐ Moderate

☐ Relevant

☐ Very relevant

4-7. Expectations for collaboration with other manufacturers

☐ Never relevant

☐ Not very relevant

☐ Moderate

☐ Relevant

☐ Very relevant

4-8. Expectations for manufacturing a generic drug

☐ Never relevant

☐ Not very relevant

☐ Moderate

☐ Relevant

☐ Very relevant

4-9. Other (please specify)

The following are general questions regarding patent linkage system in Korea.

5. Please evaluate the level of impact of patent linkage system on each of the following items.

**Enhanced research and development capability**

5-1. Research on new drugs

☐ Very low

☐ Low

☐ Moderate

☐ High

☐ Very high

5-2. Research on modified new drugs

☐ Very low

☐ Low

☐ Moderate

☐ High

☐ Very high

5-3. Research on the first generic

☐ Very low

☐ Low

☐ Moderate

☐ High

☐ Very high

5-4. Analyze related patents

☐ Very low

☐ Low

☐ Moderate

☐ High

☐ Very high

Increased sales

5-5. Domestic sales

☐ Very low

☐ Low

☐ Moderate

☐ High

☐ Very high

5-6. Overseas sales

☐ Very low

☐ Low

☐ Moderate

☐ High

☐ Very high

Improved access to medicines

5-7. Access to new drugs

☐ Very low

☐ Low

☐ Moderate

☐ High

☐ Very high

5-8. Access to generic drugs

☐ Very low

☐ Low

☐ Moderate

☐ High

☐ Very high

Increased employment

5-9. Research and development department

☐ Very low

☐ Low

☐ Moderate

☐ High

☐ Very high

5-10. Patent department

☐ Very low

☐ Low

☐ Moderate

☐ High

☐ Very high

5-11. Regulatory affairs department

☐ Very low

☐ Low

☐ Moderate

☐ High

☐ Very high

5-12. Pricing and reimbursement department

☐ Very low

☐ Low

☐ Moderate

☐ High

☐ Very high

5-13. Sales and marketing department

☐ Very low

☐ Low

☐ Moderate

☐ High

☐ Very high

The following are general questions regarding your company.

6-1. Which of the following companies does your company belong to?

☐ Multinational pharmaceutical company

☐ Domestic pharmaceutical company

6-2. Has your company been designated as an innovative pharmaceutical manufacturer?

☐ Yes

☐ No

Does your company have following experiences? Please check all relevant items.

6-3. Has your company ever applied for a patent listing in the K-Orange Book?

☐ Yes (If yes, go to question 6-4)

☐ No (If no, go to question 6-5)

6-4. Has your company ever listed a patent in the K-Orange Book?

☐ Yes

☐ No

6-5. Has your company ever filed an application for the stay of generic approval of notified drugs?

☐ Yes (If yes, go to question 6-6)

☐ No (If no, go to question 6-7)

6-6. Has your company's application for the stay of generic approval been accepted?

☐ Yes

☐ No

6-7. Has your company ever notified the registered patentee of the application for marketing approval of the drug?

☐ Yes (If yes, go to question 6-8)

☐ No (If no, go to question 6-9)

6-8. Has your company experienced the stay of generic approval for the notified drugs?

☐ Yes

☐ No

6-9. Has your company ever applied for first generic exclusivity?

☐ Yes (If yes, go to question 6-10)

☐ No (If no, go to question 6-11)

6-10. Has your company ever been granted first generic exclusivity?

☐ Yes

☐ No

6-11. Has your company ever experienced in developing new drugs?

☐ Yes

☐ No

6-12. Has your company ever experienced in developing modified new drugs?

☐ Yes

☐ No

6-13. How much is your company's annual sales?

☐ < 100,000 million KRW

☐ 100,000 or more and less than 300,000 million KRW

☐ ≥ 300,000 million KRW

☐ Other (please specify)

6-14. What extent the ratio of research and development to your company's annual sales?

☐ < 5%

☐ 5% or more and less than 7%

☐ 7% or more and less than 10%

☐ ≥ 10%

☐ Other (please specify)
